# Supplementary material for: Assay for Evaluating the Abundance of Vibrio cholerae and Its O1 Serogroup Subpopulation from Water without DNA Extraction
Source: Pathogens. 2022 Mar 16;11(3):363. doi: 10.3390/pathogens11030363 (PMC8953119; doi:10.3390/pathogens11030363)
Supplement: Supplementary file 1 [file pathogens-11-00363-s001.zip › pathogens-1617079-supplementary.pdf]

Supplementary data

Table S1. Determination of limit of detection of sample after concentrating step and testing inhibition in the assay

**CFU/ 1mL sample**	**CFU/ 500μL sample**	**CFU/ 10μL concentrate**	**Cq values with STDEV of Replicates**				
			Replicate 1	Replicate 2	Replicate 3	Mean (Cq)	STDEV
3000	1500	1500	27.0	27.0	27.0	27.0	0.01
300	150	150	30.2	30.0	30.2	30.1	0.08
30	15	15	33.5	33.7	33.5	33.6	0.09
6	3	3	35.8	34.7	35.9	35.5	0.52
5	2.5	2.5	36.7	36.9	36.8	36.8	0.09
3	1.5	1.5	No amplification				
